# Experimental phage therapy against haematogenous multi-drug resistant *Staphylococcus aureus* pneumonia in mice

**DOI:** 10.4102/ajlm.v5i1.435

**Published:** 2016-09-30

**Authors:** Joseph M. Ochieng’ Oduor, Nyamongo Onkoba, Fredrick Maloba, Atunga Nyachieo

**Affiliations:** 1 Institute of Primate Research (IPR), Nairobi, Kenya; 2 School of Medicine, Kenyatta University, Nairobi, Kenya; 3 School of Pure and Applied Sciences, Kenyatta University, Nairobi, Kenya

## Abstract

**Background:**

Community-acquired haematogenous *Staphylococcus aureus* pneumonia is a rare infection, though it can be acquired nosocomially. Currently, antibiotics used against *S. aureus* pneumonia have shown reduced efficacy. Thus, there is need for an alternative therapy against multidrug-resistant *S. aureus* (MDRSA) strains in the community.

**Objective:**

We sought to determine the efficacy of environmentally-obtained *S. aureus* lytic phage against haematogenous MDRSA pneumonia in mice.

**Methods:**

Phages and MDRSA were isolated from sewage samples collected within Nairobi County, Kenya. Isolated *S. aureus* bacteria were screened for resistance against ceftazidime, oxacillin, vancomycin, netilmicin, gentamicin, erythromycin, trimethroprim-sulfamethoxazole and cefuroxime. Thirty BALB/c mice aged six to eight weeks were randomly assigned into three groups: the MDRSA-infection group (*n* = 20), the phage-infection group (*n* = 5) and the non-infection group (*n* = 5). Mice were infected with either MDRSA or phage (108 CFU/mL) and treated after 72 hours with a single dose of clindamycin (8 mg/kg/bwt) or 108 PFU/mL of phage or a combination therapy (clindamycin and phage). The efficacy of phage, clindamycin or clindamycin with phage combination was determined using resolution of lung pathology and bacterial load in lung homogenates.

**Results:**

The viable MDRSA count was 0.5 ± 0.2 log_10_ CFU/gm in the phage-treated group, 4.4 ± 0.2 log_10_ CFU/gm in the clindamycin-treated group and 4.0 ± 0.2 log_10_ CFU/gm in the combination-treated group. The efficacy of phage therapy was significantly different from other therapeutic modes (*p* = 0 < 0.0001). Histology showed that the mice treated with phage did not develop pneumonia.

**Conclusion:**

Phage therapy is effective against haematogenous MDRSA infection. Thus, it can be explored as an alternative treatment method.

## Introduction

During lung infection with bacteria and viruses, the lung tissue becomes inflamed, deflated and occupied by pus and mucus, and the bronchial walls thicken due to accumulation of lymphocytes.^1^ *Streptococcus pneumoniae, Haemophilus influenzae, Mycoplasma pneumoniae* and *Staphylococcus aureus* all cause pneumonia.^2^ *S. aureus* pneumonia is usually associated with nosocomial infections common with patients in intensive care units.^3^

Community-acquired pneumonia, especially haematogenous (blood-borne) *S. aureus* pneumonia is rare; however, it has been documented to have high morbidity and mortality rates world-wide.^4^ Haematogenous pneumonia is common in children, immunocompromised persons and the elderly who have chronic skin and soft tissue infections.^5^ Globally, there has been an emergence of multidrug-resistant bacteria, including methicillin resistant *S. aureus* and multidrug-resistant *S. aureus* (MDRSA), which are difficult to treat with currently available antibiotics.^6,7^ These ‘super bugs’ have made the treatment of haematogenous pneumonia and other associated infections difficult.^8,9,10,11^ Thus, there is need for an alternative therapeutic measure, such as phage therapy, to combat super bugs.

Phage therapy refers to the use of bacteriophages (or phages) as antibacterial agents against bacterial pathogens.^12^ Currently, the use of phage therapy is common primarily in Georgia and Russia, despite once being practised globally before the advent of antibiotics.^13,14,15^ Its use ceased in western Europe, the United States and Canada in the 1940s due to poor understanding of the nature, safety, therapeutics and pharmacology of phages.^16^ Phage therapy is cheap in terms of development and use as a therapeutic agent. Phages are abundant in nature and are capable of *in vivo* auto-dosing, that is to say, they are capable of multiplying into several copies at the infection site; hence, only a small dose is required for treatment.^17^ In Kenya, community-acquired MDRSA infections are commonly reported among HIV patients and at public hospitals that are located in informal settlements or slums.^18^ Thus, there is a need to characterise the profile of bacterial strains that are currently circulating in environmental sources. This will provide necessary information that can be used in management of infections caused by MDRSA bacteria. This study sought to determine the therapeutic potential of environmentally-obtained lytic phages in Nairobi, Kenya, against haematogenous MDRSA pneumonia in BALB/c mice.

## Research method and design

### Ethical considerations

All experimental protocols and procedures of the study were reviewed and approved by the Institutional Review Committee on Animals Ethics of the Institute of Primate Research (ref no: IRC/02/14) ethical committee on the use of laboratory animals for biomedical science in accordance with the international guidelines on animal care, handling and use for biomedical research. The experiments are reported in accordance with ARRIVE guidelines.^19^

### Study animals

The study included 30 BALB/c mice of mixed sexes aged 6 to 8 weeks which were sourced from the Institute of Primate Research rodent house, Karen-Nairobi, Kenya, and maintained at their rodent facility. The mice were fed on antibiotic-free food rations (obtained from Unga Feeds, Nairobi, Kenya) and water was provided *ad libitum*.

### Isolation of MDRSA

*S. aureus* bacterial strains were isolated from 20 sewage samples collected from informal settlements, sewage treatment plants and abattoirs within Nairobi County, Kenya. Specimens from all the samples were streaked on selective Mannitol salt agar (Liofilchem^®^, Roseto degli Abruzzi, Italy) supplemented with 4 µg of ciprofloxacin [Liofilchem^®^, Roseto degli Abruzzi, Italy]. Single colonies were amplified in nutrient agar (HiMedia, Mumbai, India). *Staphylococcus* strains were identified using microscopy, physiological tests and an Analytical Profile Index of *Staphylococcus* system [bioMérieux, Marcy l’Etoile, France]. We screened isolated *S. aureus* bacteria for antimicrobial resistance to the following drugs: ceftazidime 30 µg, oxacillin 1 µg, vancomycin 30 µg, netilmicin 30 µg, gentamicin 10 µg, erythromycin 15 µg, trimethroprim-sulfamethoxazole 25 µg and cefuroxime 30 µg (Liofilchem^®^, Roseto degli Abruzzi, Italy), according to the Clinical Laboratory Standard Institute protocol.^20^ Isolates were considered to be MDRSA, if they were non-susceptible to more than one class of antibiotics. Colonies identified as MDRSA were re-suspended in 50% glycerol-nutrient broth and stored at -20 ^°^C until use.

### Phage isolation

We analysed half a litre of sewage samples collected from 10 sites (Nairobi City sewage treatment plant, informal settlements and an abattoir) within Nairobi County, Kenya. Twenty specific *S. aureus* lytic phages were isolated from these samples using the modified method of Anany et al.^21^ Briefly, equal volumes of ultra-filtered sewage samples and nutrient broth, plus 1 mL of 18-hour-old MDRSA culture were mixed and incubated overnight at 37 ^°^C while shaking at 120 rpm (LAB-LINE^®^ INCUBATOR-SHAKER, Waltham, Massachusetts, United States). The following day, the culture was centrifuged at 10 000 g for 10 minutes (Fisher Centrific^®^ Centrifuge, Waltham, Massachusetts, United States), and the supernatant was filtered through a 0.22 µm filtration unit (Cambridge, Massachusetts, United States), then screened for the presence of phages using a double-layer plaque assay. Individual plaques were selected and sub-cultured in 2 mL of nutrient broth containing a sensitive bacterial host (10^6^ CFU/mL). The isolated phages were then tested against the previously-isolated MDRSA by spot assay. The virulence of the phages was determined by using an efficiency of plating analysis,^22^ whereby the strain with numerous plaques on MDRSA lawn was considered the most virulent. The virulent phage was selected and used as a therapeutic agent against MDRSA in mice.

### Experimental design

**FIGURE 1:**
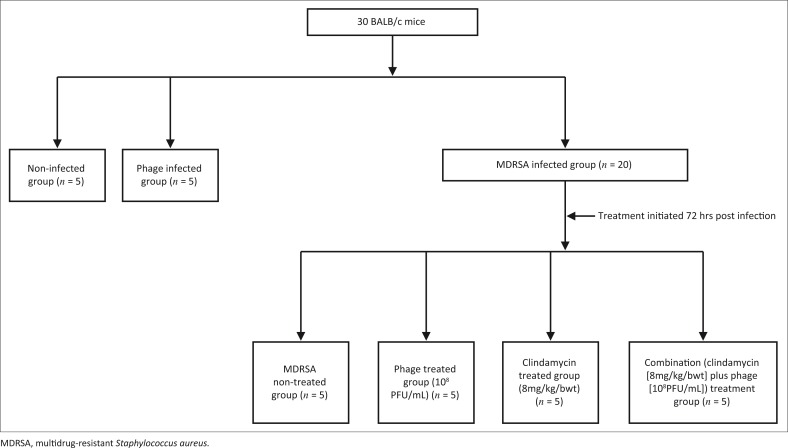
Design of the *in vivo* experimental study. The study was repeated three times and a total number of 90 mice were used.

Thirty mice were randomly assigned to one of three groups: the MDRSA infection group (*n* = 20), the non-infection group (*n* = 5) and the phage infection group (*n* = 5). Twenty mice were infected with 10^8^ CFU/mL of MDRSA isolate intravenously by means of the tail vein and then sub-divided into four sub-groups: MDRSA with clindamycin (Clindar, Indi Pharma, Mumbai, India) (8 mg/kg body weight) treatment (*n* = 5); MDRSA with phage (10^8^ PFU/mL) treatment (*n* = 5); MDRSA with combination treatment (clindamycin [8 mg/kg body weight] and phage [10^8^ PFU/mL]) (*n* = 5); and MDRSA with no treatment as the control (*n* = 5) (Figure 1). Presence of infection was determined by observing the physical appearance of the mice and assessing the bacteraemia level. The bacteraemia count was assessed by culturing blood samples from each mouse by using the pour plate method. Fifty microlitres (50 µL) of blood was obtained from each mouse from the tail vein and mixed with 950 µL sterile normal saline. This mixture (blood-normal saline) was added to molten nutrient broth, allowed to cool, then incubated overnight at 37 ^°^C. The bacterial colony count was recorded per millilitre for each mouse. A similar procedure was used to determine the bacteraemia for each mouse after treatment was initiated. Treatment with clindamycin, phage or combination was administered at 72 hours post-MDRSA infection when the bacterial levels in the blood began to rise. Each mouse in the treatment groups received a single dose of the mentioned therapeutic agents. The experiments were repeated three times; thus, the total number of mice used in the study was 90.

### Viable bacteria levels in mouse lung homogenates

Upon necropsy, lungs were obtained aseptically and homogenised for bacterial culture as follows: 500 µL of homogenised tissue was diluted with normal saline at a ratio of 1:20 (homogenised lung tissue to saline). The diluted, homogenised tissue was then plated on 7.5% sodium chloride nutrient agar to select for MDRSA and incubated at 37 ^°^C for 18–20 hours.

### Histopathology

Phage safety and MDRSA pathogenicity were determined by euthanising the mice when they exhibited poor physical appearance and breathing difficulties in order to alleviate pain and suffering. Surviving mice were euthanised at 10 days post-infection. Lung samples were collected in 10% formaldehyde for histological analysis. Levels of inflammation were scored using categories as described by Schünemann *et al*.^23^ Briefly, the categories worst, worse, bad, good, better and best were assigned based on a scale of 5 to 0. The ‘worst’ category was assigned for severely inflammed septa, numerous collapsed alveoli, large pockets of pneumonia, mucus-congested alveoli, presence of perivascular fibrosis blood vessels and lack of ventilation (score = 5). The ‘worse’ category included all conditions in the worst category but with poor ventilation (score = 4). The ‘bad’ category was assigned for minor inflammation of septa, few pockets of pneumonia and moderate ventilation (score = 3). The ‘good’ category was assigned for minor inflammation of septa, few pockets of mucus-congested alveoli and good ventilation (score = 2). The ‘better’ category was assigned for no inflammed septa and a few pockets of mucus-congested alveoli (score = 1). The ‘best’/normal category was assigned for no inflammation and good ventilation (score = 0).

### Statistical analysis

Bacterial and phage counts were represented as mean ± standard error of the mean. The statistical significance of differences between groups was determined by one-way analysis of variance, followed by Tukey’s multiple comparison test, using Graph Pad Prism 5.0.1 (Graph pad software, San Diego, California, United States). A *p*-value of less than 0.05 was considered statistically significant.

## Results

### Multidrug-resistant S. aureus isolation

**FIGURE 2:**
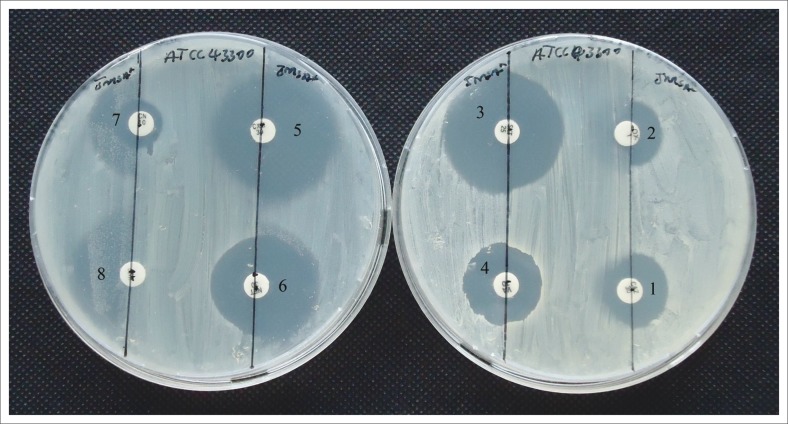
Antibiogram test of *S. aureus* isolate. The isolate was resistant to: ceftazidime, 30 µg (1); oxacillin, 1 µg (2); vancomycin, 30 µg (4); netilmicin, 30 µg (6); gentamicin, 10 µg (7); erythromycin, 15 µg (8); and cefuroxime, 30 µg (5). However, it was sensitive to trimethroprim-sulfamethoxazole, 25 µg (3).

The environmentally-isolated *S. aureus* bacterium was resistant to *β*-lactam antibiotics (ceftazidime, cefuroxime and oxacillin). In addition, it was non-susceptible to glycopeptide (vancomycin), aminoglycoside (netilmicin and gentamicin) and macrolide (erythromycin) antibiotics. However, it was susceptible to nucleic acid synthesis inhibitors (co-trimoxazole [trimethroprim-sulfamethoxazole]) (Figure 2). Thus, the bacterial isolate was considered to be multidrug-resistant, as it was non-susceptible to more than one class of antibiotics.

### Phage isolation and *in vitro* activities

**FIGURE 3:**
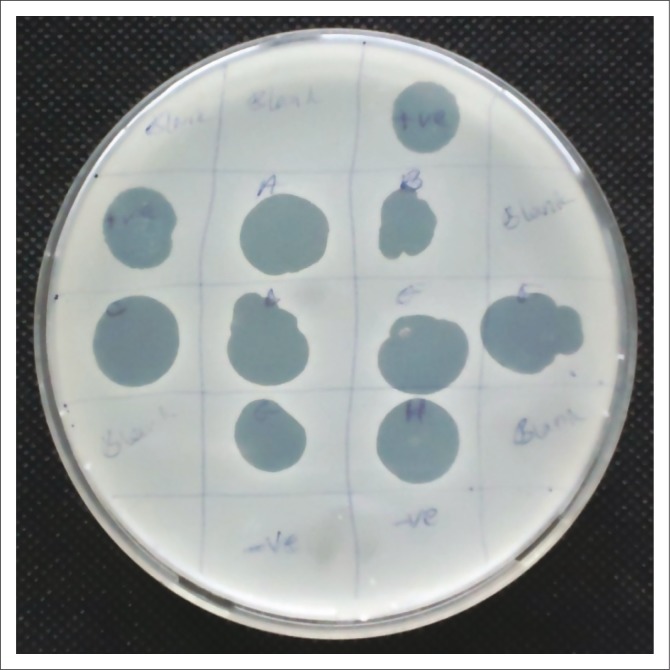
Spot assay showing most virulent phage isolate.

Spot assays were used to identify 10 *S. aureus* lytic phages which produced large clear plaques on the MDRSA lawn (Figure 3) while other isolates failed. Of the 10 phage isolates, one created a larger patch on the MDRSA lawn. Its efficiency of plating analysis showed that it had more plaques than other phages.

### Therapeutic effects in mice

Two groups of mice had 100% survivorship (*n* = 5), namely, the non-infected/non-treated group and the phage-infected mice. Only three mice survived in the MDRSA-infected group before treatment (72 hours post-infection) commenced. All the mice that were treated with either clindamycin, phage or the combination therapy (clindamycin plus phage) survived at day 7 post-infection and each group had three mice. Mice that had phage infection (phage control group) were more active than mice in other treatment groups (clindamycin, phage and combination). Only one mouse in the MDRSA-infected control group survived at day 7 post-infection and it was weak (Table 1).

**TABLE 1:** Number of surviving mice at 72 hours post-infection and 7 days post-treatment, Nairobi, Kenya.

Groups	Initial number of mice	Number of mice 72 hours post-infection	Number of mice during treatment	Number of mice 7 days post-infection (end point)
All MDRSA infected mice	20	12	12	10
Non-treated	5	3	3	1
Clindamycin-treated	5	3	3	3
Phage-treated	5	3	3	3
Combination-treated	5	3	3	3
Phage-infected mice	5	5	5	5
Non-infected mice	5	5	5	5

**Total**	**30**	**22**	**22**	**20**

MDRSA, multidrug-resistant *Staphylococcus aureus*.

### Viable bacteria levels in mouse lung homogenates

**FIGURE 4:**
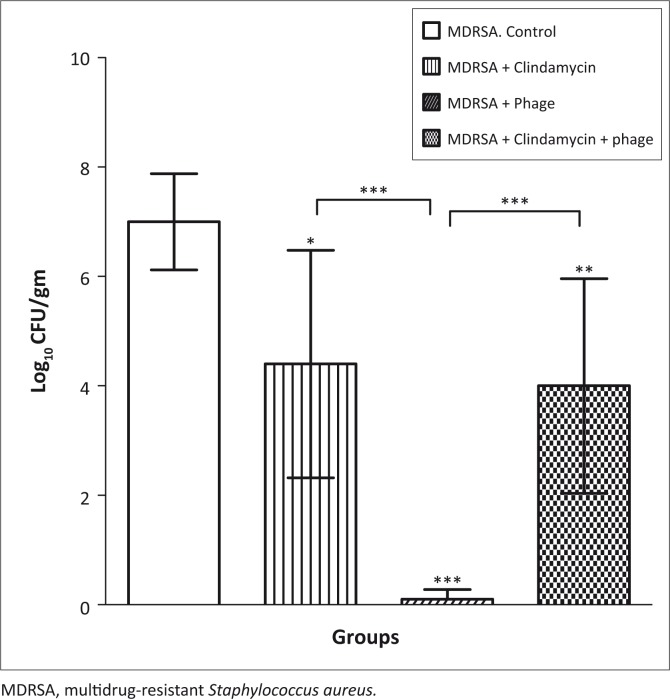
Mean bacterial count (mean Log_10_ CFU/gm ± SE) in lung tissue from MDRSA-infected mice. Controls received no treatment, remaining infected mice were treated with clindamycin, phage, or a combination therapy (clindamycin plus phage). Each group had five mice. Levels of significance: *p < 0.05; **p < 0.001; and ***p < 0.0001; each was compared with MDRSA control.

The number of viable MDRSA in mouse lung homogenates was significantly lower in mice treated with phage only (log_10_ CFU/gm = 0.5 ± 0.2) compared with those in the MDRSA-infected, non-treated control group (log_10_ CFU/gm = 8.0 ± 0.2) (*p* < 0.0001) (Figure 4). Compared with the MDRSA-infected, non-treated group, statistically-significantly differences were observed in the viable bacterial counts in the MDRSA-infected, clindamycin-treated (log_10_ CFU/gm = 4.4 ± 0.2; *p* < 0.05) and MDRSA-infected, combination-treated groups (log_10_ CFU/gm = 4.0 ± 0.2; *p* < 0.001). The differences in counts between the phage-treated group and groups treated with clindamycin and the combination therapy were also statistically significant (*p* < 0.0001).

### Histopathological examination

**FIGURE 5:**
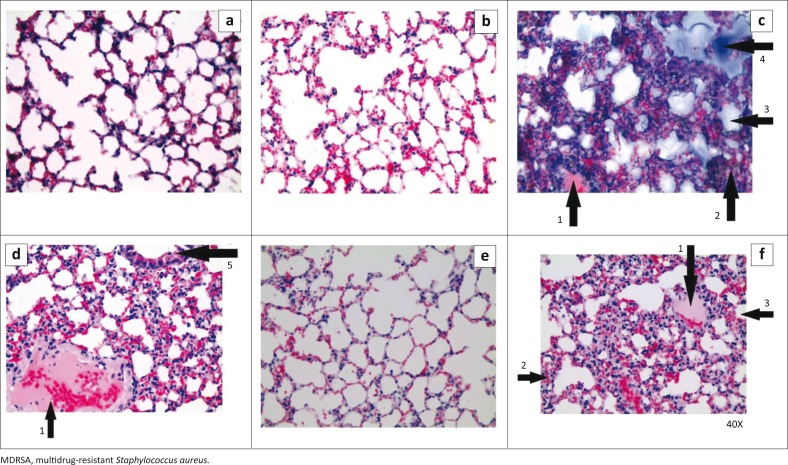
Mouse lung tissue slides (40x magnification). Tissue from non-infected, non-treated mice (A) and phage-infected, non-treated mice (B) were well ventilated. Tissue from MDRSA-infected, non-treated control mice (C) had pockets of serous fluid (pneumonia) (1), lymphocyte-infiltrated (inflamed) septa (2), deflated alveoli (3) and alveoli full of mucus (4). Tissue from MDRSA-infected, clindamycin-treated mice (D) had pockets of pneumonia (1), inflamed septa and perivascular fibrosis of blood vessels (5). Tissue from MDRSA-infected, phage-treated mice (E) was well ventilated with resolved inflammation. Tissue from MDRSA-infected mice treated with a combination of clindamycin and phage (F) had pockets of pneumonia (1), inflamed septa (2) and deflated alveoli (3).

The lung tissues of mice in the phage-infected group were relatively normal, with minor focal congestion compared with tissue from mice in the MDRSA-infected non-treated group, which had deflated alveoli congested with mucus, lymphocyte-infiltrated septa and pockets of serous fluid. The lung tissue of MDRSA-infected, non-treated mice were similar to lung tissue from clindamycin-treated mice. However, lung tissue from the clindamycin-treated group (*n* = 3) overall was graded as bad, since they had less congestion, fewer pockets of serous fluid and deflated alveoli compared with the non-treated MDRSA infected mice. Mice that received the combination therapy (*n* = 3) showed inflammation of the septa and blood vessel walls, moderately deflated alveoli and several serous fluid pockets compared with the non-infected (*n* = 5) and phage-infected mice (*n* = 5) (*p* < 0.0001) (Figure 5).

## Discussion

This study found that *S. aureus* isolates collected from sewage water and found to be resistant to multiple drug therapies caused haematogenous pneumonia in mice, which was characterised by infiltration of immune cells and accumulation of serous fluid in the lungs. This confirmed that the isolated bacterium was pathogenic and able to cause sepsis, then was further confirmed by isolation of viable MDRSA bacteria from the lung homogenates. The accumulation of serous fluid and pus in the lungs indicated that the mice induced an innate immune response toward multiplying MDRSA bacteria.^24^ This inflammatory immunological phenomenon may have led to the inflammation observed in the bronchi, bronchioles, alveoli and septa, as well as blood vessels. Our findings agree with other studies in which mice were infected intravenously with methicillin-resistant *S. aureus*, then administered a dose of antibiotic on a daily basis for a week.^25,26,27^ However, in our study we used a single dose of *S. aureus*-specific lytic phage.

The use of phage as a treatment method attained 100% efficacy, an indication that the single dose administered was enough to clear the bacteria from the infected mice. This is probably due to the auto-dosing properties of phages, whereby they increase in numbers at the site of an infection.^28^ The mode of phage administration, concentration, dosage and timing of treatments and resistance to phages by the pathogenic bacteria are some of the factors that determine the efficacy and safety of phage therapy.^29^ These factors may be responsible for the differences between our findings and those of other studies,^30,31^ in which methicillin-resistant *S. aureus*-infected mice were treated immediately or six hours after infection with phage to achieve a 100% efficacy. The timing of the initiation of treatment may be the crucial factor in achieving 100% efficacy. However, we cannot rule out the role of immunity in clearing the bacteria, considering that Yao et al.^32^ established that within 72 hours of infection the mouse immune system has the capacity to clear bacteria from the system. In our study, the combination therapy (clindamycin plus phage) reduced the efficacy of the phage to about 60%, indicating that clindamycin may have an antagonist effect on phage lytic activity. Clindamycin is a bacteriostatic antibiotic; thus, it may interfere with phage protein synthesis, which would interrupt the phage auto-dosing mechanism.

### Limitations of the study

Our study results were generated in mice. While our findings provide vital information on the antimicrobial resistance profile in Kenya, they cannot be translated directly to humans. However, comparative animal models can be utilised to ascertain the safety and therapeutic potential of environmentally-available phages against infections caused by multidrug-resistant bacteria.

### Conclusion

In conclusion, we report for the first time that a single dose of MDRSA-specific lytic phage is efficacious against haematogenous pneumonia caused by multidrug-resistant *S. aureus* in mice.
